# Bone Nanomechanical Properties and Relationship to Bone Turnover and Architecture in Patients With Atypical Femur Fractures: A Prospective Nested Case‐Control Study

**DOI:** 10.1002/jbm4.10523

**Published:** 2021-08-12

**Authors:** Lanny V. Griffin, Elizabeth Warner, Saroj Palnitkar, Shijing Qiu, Mahalakshmi Honasoge, Shawna G. Griffin, George Divine, Sudhaker D. Rao

**Affiliations:** ^1^ California Polytechnic State University (Cal Poly) San Luis Obispo CA USA; ^2^ Bone & Mineral Research Laboratory Henry Ford Health System/Wayne State University Integrative Biosciences (IBio) Research Facility Detroit MI USA; ^3^ Division of Endocrinology, Diabetes, and Bone & Mineral Disorders Henry Ford Health System Detroit MI USA; ^4^ Department of Public Health Sciences Henry Ford Health System Detroit MI USA

**Keywords:** ATYPICAL FEMUR FRACTURE, BISPHOSPHONATE, BONE BIOPSY, NANOINDENTATION, SEVERELY SUPPRESSED BONE TURNOVER

## Abstract

Atypical femur fractures (AFFs) are well‐established serious complication of long‐term bisphosphonate and denosumab therapy in patients with osteopenia or osteoporosis. To elucidate underlying mechanism(s) for the development of AFF, we performed a nested case‐control study to investigate bone tissue nanomechanical properties and prevailing bone microstructure and tissue‐level remodeling status as assessed by bone histomorphometry. We hypothesized that there would be differences in nanomechanical properties between patients with and without AFF and that bone microstructure and remodeling would be related to nanomechanical properties. Thirty‐two full‐thickness transiliac bone biopsies were obtained from age‐ and sex‐matched patients on long‐term bisphosphonate therapy with (*n* = 16) and without an AFF (*n* = 16). Standard histomorphometric measurements were made in each sample on three different bone envelopes (cancellous, intracortical, and endosteal). Iliac bone wall thickness was significantly lower on all three bone surfaces in patients with AFF than in those without AFF. Surface‐based bone formation rate was suppressed similarly in both groups in comparison to healthy premenopausal and postmenopausal women, with no significant difference between the two groups. Nanoindentation was used to assess material properties of cortical and cancellous bone separately. Elastic modulus was higher in cortical than in cancellous bone in patients with AFF as well as compared to the elastic modulus of cortical bone from non‐AFF patients. However, the elastic modulus of the cancellous bone was not different between AFF and non‐AFF groups or between cortical and cancellous bone of non‐AFF patients. Resistance to plastic deformation was decreased in cortical bone in both AFF and non‐AFF groups compared to cancellous bone, but to a greater extent in AFF patients. We conclude that long‐term bisphosphonate therapy is associated with prolonged suppression of bone turnover resulting in altered cortical remodeling and tissue nanomechanical properties leading to AFF. © 2021 The Authors. *JBMR Plus* published by Wiley Periodicals LLC on behalf of American Society for Bone and Mineral Research.

## Introduction

Atypical femur fractures (AFFs) are well‐established, serious, and life changing complication of long‐term bisphosphonate or denosumab therapy in patients with osteopenia or osteoporosis.^(^ ^1^, ^2^, ^3^, ^4^, ^5^, ^6^, ^7^, ^8^ ^)^ However, the scope, magnitude, and pathogenesis of these unusual fractures (often referred to as AFF) remains to be established. In 2005, we proposed that severe suppression of bone turnover (SSBT) is a major contributing factor to the development of AFF,^(^ ^1^, ^2^ ^)^ which was later confirmed by others.^(^ ^9^, ^10^, ^11^ ^)^ However, since our initial proposal, we have found that many patients with SSBT, as we defined at the time, do not necessarily develop AFF,^(^ ^12^ ^)^ and conversely, not all patients with AFF necessarily manifest SSBT on bone histomorphometry.^(^ ^11^, ^12^, ^13^, ^14^ ^)^ The apparent conflicting observations imply that factors, in addition to SSBT, must be contributing to the pathogenesis of unusual fractures including AFFs.^(^ ^15^ ^)^

Several investigators have proposed various risk factors for the development of AFF such as race/ethnicity,^(^ ^16^, ^17^, ^18^ ^)^ younger age,^(^ ^19^ ^)^ sex,^(^ ^20^ ^)^ femur geometry or generalized femoral cortical thickening,^(^ ^21^, ^22^ ^)^ duration and type of bisphosphonate therapy,^(^ ^5^, ^20^, ^23^ ^)^ drugs known to suppress bone turnover,^(^ ^24^, ^25^ ^)^ certain comorbid conditions,^(^ ^26^ ^)^ and SSBT.^(^ ^1^, ^9^, ^10^, ^11^ ^)^ Of all the risk factors identified, only the generalized cortical thickness has not been confirmed in independent studies.^(^ ^27^, ^28^ ^)^ A few studies have examined mechanical and compositional properties of bone in both ex vivo,^(^ ^29^, ^30^, ^31^ ^)^ and in vivo^(^ ^32^, ^33^ ^)^ experiments, including our recent study,^(^ ^34^ ^)^ and the results are conflicting. In our previous small cohort studies, we found significant differences in nanomechanical and compositional properties in iliac bone from patients with AFF compared to both bisphosphonate‐naive osteoporotic patients and normal healthy non‐osteoporotic subjects.^(^ ^29^, ^30^, ^31^ ^)^ However, these studies lacked appropriate control bone biopsy specimens from patients treated with long‐term bisphosphonate therapy, who had not sustained an AFF. To elucidate the underlying mechanism(s) for the development of AFF, we designed a nested case‐control study to assess bone tissue nanomechanical properties in postmenopausal women receiving long‐term bisphosphonate therapy for osteoporosis and relate these properties to the prevailing bone microstructure and tissue‐level remodeling as assessed by standard bone histomorphometry.^(^ ^35^ ^)^ We hypothesized that there would be differences in the nanomechanical properties between patients with and without an AFF and that the bone microstructure and remodeling would be related to the tissue‐level nanomechanical properties.

## Patients and Methods

### Patient and bone biopsy selection

Thirty‐two postmenopausal women with osteoporosis who were on long‐term bisphosphonate therapy (>2 years) were selected from a larger pool of 80 patients (20 with AFF and 60 without AFF), who underwent transiliac bone biopsies between 2014 and 2018 as part of the parent study (Pathogenesis of Atypical Femur Fractures; NCT02155595; https://clinicaltrials.gov/ct2/show/NCT02155595). All patients were recruited consecutively without any ascertainment bias and the interval between AFF diagnosis and biopsy was <6 months. The 32 transiliac bone biopsies with intact cortices were included in this study (16 from patients with AFF and 16 from age‐, sex‐, and race‐matched patients with no AFF; Table 1). Of the 32 patients, 30 were treated with alendronate and one each with zoledronic acid and denosumab at standard clinical therapeutic doses and frequency. Complete AFF were confirmed by x‐rays and incomplete AFF were confirmed by single‐energy femur scanning on a Hologic bone densitometer (Marlborough, MA, USA) and by digital tomosynthesis of femurs as appropriate. The study was approved by the Institutional Review Board at Henry Ford Hospital and a written informed consent was provided by all the participants.

**Table 1:** Demographic Data of Patients With and Without an AFF

Characteristic	Patients without an AFF	Patients with an AFF
Sample size, *n*	16	16
Female (%)	100	100
Race, *n*		
Asian	0	1
Black	2	2
White	14	13
Age (years), mean ± SD	68.1 ± 6.6	68.2 ± 7.4
BP treatment duration (years), mean ± SD	7.5 ± 4.7	11.5 ± 4.9
Fracture morphology, *n*		
Complete AFF		16
Incomplete AFF		8
Bilateral		8
Unilateral		8

Female percentage is 100% by design (please see text for details). Total for complete and incomplete AFFs (*n* = 24) exceeds total sample size (*n* = 16) because 8 patients had bilateral involvement (3 with bilateral complete AFF, 2 with bilateral incomplete AFF, and 3 with both type in each femur).

BP = bisphosphonate.

### Bone histomorphometry

Before biopsy, all patients received in vivo double tetracycline labeling with an interlabel interval of 14 days. The transiliac bone biopsies with intact cortices were obtained using a 7.5‐mm trephine (Rochester Bone Biopsy Trephine; Medical Innovations International, Inc., Rochester, MN, USA) and were processed, embedded, sectioned, stained, and examined as reported.^(^ ^36^ ^)^ To reduce variability in sample procurement, all biopsies were performed by a single operator (SDR). To reduce bias, all bone samples were measured by a single histotechnologist (SP) who was unaware of the patient information (AFF and bisphosphonate treatment status). All bone histomorphometric variables are designated in accordance with the nomenclature recommended by the American Society for Bone and Mineral Research.^(^ ^35^ ^)^

The static histomorphometric indices were measured in sections stained with modified Toluidine blue, and the dynamic remodeling indices were measured in unstained sections. All the measurements were performed using a Bioquant image analysis system (Nashville, TN, USA) equipped with a bright‐field and fluorescence microscope. The parameters related to bone structure included fraction of total bone volume per tissue volume (BV/TV, %), trabecular thickness (Tb.Th, μm) and number (Tb.N, 1/mm^2^), and cortical thickness (Ct.Th, μm). Static and remodeling indices were measured separately on the cancellous, intracortical, and endosteal surfaces. The static indices included osteoid and eroded surfaces as a fraction of bone surface (OS/BS, %; ES/BS, %), wall thickness (W.Th, μm), and osteoid thickness (O.Th, μm). The surface lengths covered by osteoblasts and osteoclasts (Ob.S and Oc.S) were measured separately and expressed as a fraction of bone surface (Ob.S/BS, %; Oc.S/BS, %).

The dynamic remodeling indices were measured based on tetracycline labeling. The extent of bone mineralizing surface (MS) was labeled by double or single tetracycline labeling, from which the MS as a fraction of total bone surface (MS/BS, %) was calculated. Mineral apposition rate (MAR, μm/day) was obtained from the average distance between the two tetracycline labels divided by the interval of administration (14 days in our study). Bone formation rate at the surface level (BFR/BS, μm^3^/μm^2^/year) was calculated as MAR*(MS/BS). Activation frequency (Ac.f, #/year), the annual probability of activation of a new remodeling site at any given locus on the bone surfaces, was derived from BFR/BS)/W.Th. For surfaces containing only a single label, a minimum value of 0.3 μm/day was assigned to MAR; if there was no label, the MAR was treated as a missing value, and MS/BS, BFR/BS and Ac.f were assigned a value of zero.^(^ ^12^, ^37^ ^)^

### Nanoindentation

**Fig. 1:**
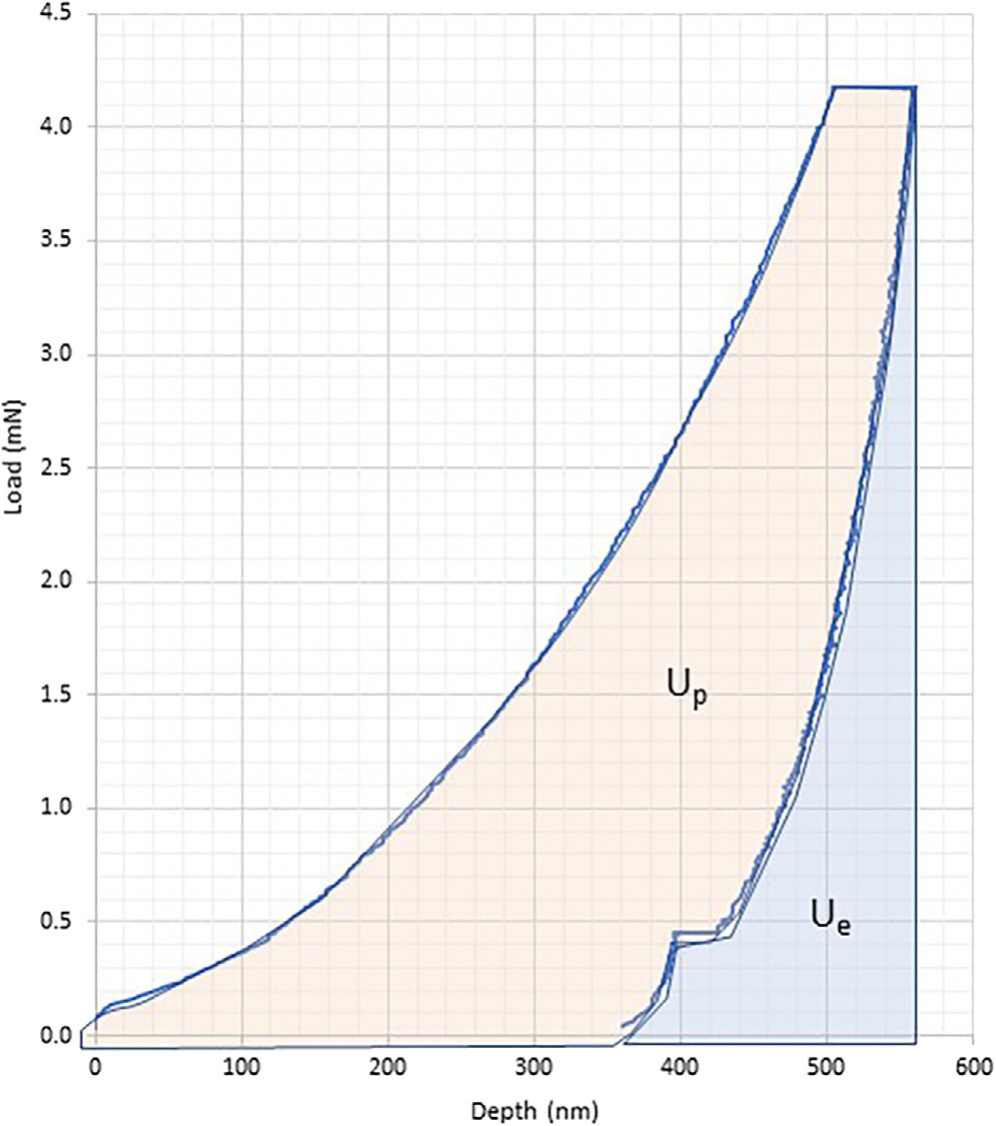
A typical indentation curve. The elastic (Ue) and plastic energy (Up) are denoted on the indentation curves.

The embedded bone biopsies were prepared for nanoindentation by polishing to 0.05‐μm standard metallographic techniques. A nanoindenter (NanoTest 600; Micromaterials, LTD, Wrexham, UK) was used to measure the force and displacement during indentation of the polished bone specimen. Nanoindentation was performed using a Berkovich diamond indenter tip (*E*_i_ = 1141 Gpa, *v*_i_ = 0.07, where *E*_i_ represents elastic modulus and *v*_i_ represents Poisson's ratio of the indenter). The indentation procedure was performed under displacement control. After the surface was identified, the indenter was advanced to 500 nm at a loading rate of 0.25 mN/s. The indentation included a 60‐second holding period at maximum load to account for creep and a 100‐second holding period for thermal drift at 10% of maximum load. For each specimen, 60 sites were measured in cortical bone, and 12 sites were measured in each of five trabeculae. The measurement areas were determined using an optical microscope at magnification ×400. Bone tissue elastic modulus (*E*) and contact hardness (*H*_*c*_) were calculated using a mathematical solution derived by Oliver and Pharr^(^ ^38^ ^)^ and resistance to plastic deformation (H) was calculated using previously described methods.^(^ ^29^, ^30^ ^)^ The elastic energy (U_e_), defined as the amount of indentation energy recovered, is calculated as the area under the unloading curve (AUC). The plastic energy (U_p_), defined as unrecoverable energy spent on processes such as microcracking, was calculated by subtracting the elastic energy from the total energy (Fig. 1).

### Statistical analysis

Bone histomorphometric variables are expressed as mean ± SD and differences between patients with and without AFF were compared by unpaired *t* test. Nanoindentation results, elastic modulus (E), contact hardness (H_c_), resistance to plastic deformation (H), elastic energy (U_e_), and plastic energy (U_p_), are reported as mean ± SD or 95% CI.

A mixed effects logistic regression model was used to assess the significance of explanatory variables (treatment duration, mechanical property) on the binary outcome: AFF or non‐AFF (Proc GEE, version 9.4; SAS Institute, Inc., Cary, NC, USA). Cortical and trabecular bone components were analyzed separately. Odds ratios (ORs) were calculated for treatment duration and nanoindentation property. Differences with a *p* value <0.05 were considered significant.

## Results

All patients in this substudy were women by design of the parent protocol (NCT02155595). Of the 32 women, four were black, one was Asian, and 27 were white. The mean age of the cohort was 68.1 ± 6.9 years; 31 were treated with bisphosphonates (alendronate = 30 and zoledronic acid = 1) and one was treated with denosumab for at least ≥2 years (Table 1). Although the mean age of the two groups (women with AFF versus women with no AFF) was identical (mean 68 years) because of matching, patients with an AFF had been on antiresorptive therapy for a significantly longer duration (11.6 ± 4.9 years; range, 2 to 20 years) than non‐AFF patients (7.4 ± 4.8 years; range 2 to 20 years; *p* = 0.006). None of the patients were on long‐term corticosteroids, pharmacologic high‐dose vitamin D, or any other concurrent antiresorptive therapy, except one patient who was receiving estrogen with alendronate. Four patients had diabetes mellitus (three in AFF and one in non‐AFF group), and none had conditions or taking medications known to cause osteoporosis.

### Histomorphometry

Relevant bone histomorphometric data are summarized in Table 2. The differences in structural variables, including cancellous bone volumes (Cn‐BV/TV), trabecular thicknesses (Tb.Th), trabecular number (Tb.N), cortical bone volume (Ct‐BV/TV), and cortical thickness (Ct.Th), were not significant between AFF and non‐AFF patients. For static variables, no bone envelope showed significant difference in erosion surface (ES/BS), osteoid surface (OS/BS), osteoid thickness (O.Th), or osteoblast surface (Ob.S/BS) between the two groups. Wall thickness (W.Th) in all three bone envelopes was significantly lower in patients with AFF than in non‐AFF patients. However, osteoclast surface (Oc.S/BS) in the intracortical envelope was significantly higher in AFF patients compared to non‐AFF patients. Tetracycline‐labeled dynamic variables showed that mineral apposition rate (MAR), bone formation rate at surface level (BFR/BS), and activation frequency (Ac.f) were not significantly different in all bone envelopes between the two groups. However, the degree of suppression of both BFR/BS and Ac.f were similar in AFF and non‐AFF patients as compared to normal postmenopausal women without osteoporosis.^(^ ^36^ ^)^

**Table 2:** Relevant Bone Histomorphometric Data

	Cancellous bone			Intracortical bone			Endosteal bone		
Parameter	non‐AFF	AFF	*p*	non‐AFF	AFF	*p*	non‐AFF	AFF	*p*
BV/TV (%)	15.2 ± 5.10	14.1 ± 5.47	0.587	95.3 ± 1.41	93.9 ± 3.75	0.619			
Tb.Th (μm)	107 ± 24.5	103 ± 25.9	0.462						
Tb.N (#/mm ^2^ )	1.39 ± 0.287	1.33 ± 0.235	0.551						
Ct.Th (mm)				0.987 ± 0.278	1.02 ± 0.302	0.772			
W.Th (μm)	33.6 ± 3.69	29.8 ± 4.61	0.018	42.6 ± 4.66	36.1 ± 4.22	<0.001	37.2 ± 4.36	33.3 ± 5.50	0.010
ES/BS (%)	2.10 ± 1.80	3.62 ± 3.22	0.158	2.26 ± 2.78	3.57 ± 1.97	0.149	3.44 ± 3.70	7.44 ± 8.66	0.140
Oc.S/BS (%)	0.298 ± 0.328	0.979 ± 1.21	0.062	0.288 ± 0.479	0.567 ± 0.342	0.002	0.604 ± 0.932	2.19 ± 2.72	0.133
OS/BS (%)	1.96 ± 2.42	5.21 ± 7.70	0.229	5.10 ± 3.98	8.09 ± 6.35	0.245	5.18 ± 4.39	7.75 ± 7.20	0.157
O.Th (μm)	8.08 ± 5.00	7.40 ± 3.18	0.663	6.51 ± 2.34	7.40 ± 3.32	0.402	5.38 ± 3.40	5.87 ± 2.95	0.856
Ob.S/BS (%)	0.357 ± 0.697	1.05 ± 1.63	0.334	0.867 ± 1.16	1.77 ± 2.22	0.321	0.803 ± 0.733	2.41 ± 3.35	0.568
MAR (μm/day)	0.323 ± 0.148	0.359 ± 0.179	0.676	0.331 ± 0.216	0.356 ± 0.139	0.735	0.311 ± 0.263	0.268 ± 0.129	0.822
BFR/BS (μm ^3^ /μm ^2^ /year)	1.51 ± 2.35	2.01 ± 2.81	0.708	5.02 ± 5.36	5.10 ± 6.23	0.663	2.63 ± 3.58	3.36 ± 5.43	0.781
Ac.f (#/year)	0.042 ± 0.061	0.064 ± 0.090	0.967	0.115 ± 0.124	0.150 ± 0.189	0.983	0.074 ± 0.103	0.104 ± 0.164	0.881

Data are expressed as mean ± SD.

### Nanoindentation

**Fig. 2:**
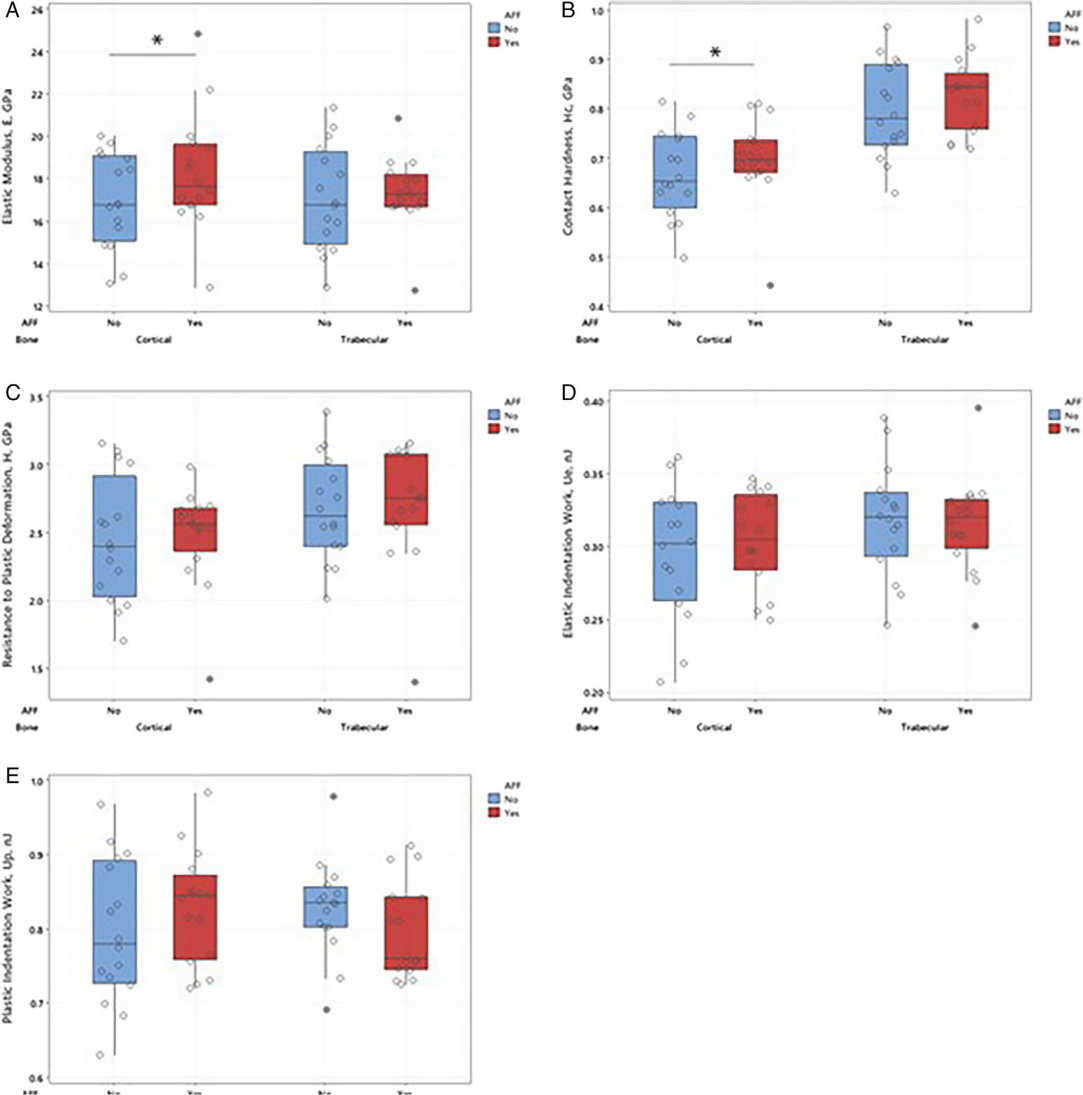
Average nanoindentation data for cortical and trabecular bone with and without an atypical femur fracture. The data are presented as mean with 95% confidence intervals for elastic modulus (*A*), resistance to plastic deformation (*B*), contact hardness (*C*), elastic indentation work (*D*), and plastic indentation work (*E*). Differences with an asterisk are statistically significant at *p* < 0.05.

**Fig. 3:**
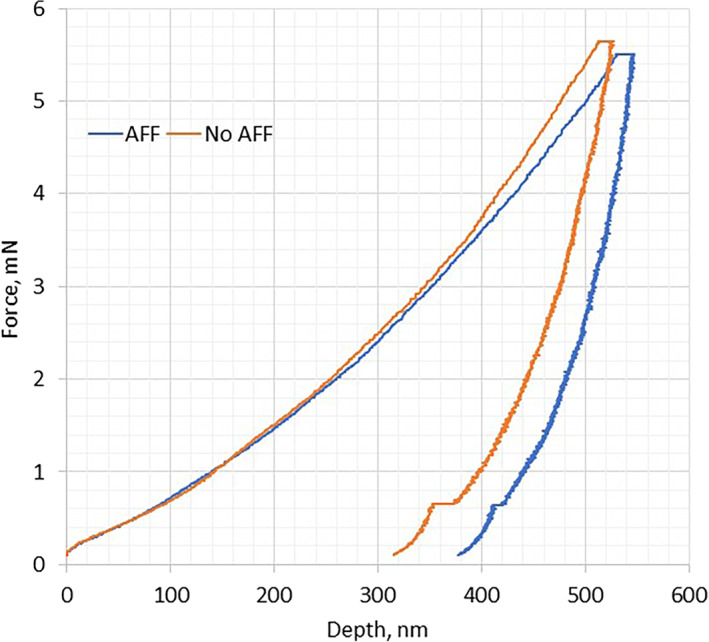
Typical average force‐depth curves for bone with and without an atypical femur fracture. For the curve representing no‐AFF, E = 16.2 GPa, H = 1.178 GPa, Hc = 0.77 GPa, Ue = 0.44 nJ, Up = 0.82 nJ. For the curve representing an AFF, E = 18.6 GPa, H = 1.63 GPa, Hc = 0.56 GPa, Ue = 0.37 nJ, Up = 0.91 nJ.

Relevant cortical and cancellous bone tissue nanoindentation results are depicted in Fig. 2 and representative force‐depth curves for an AFF and non‐AFF subjects are in Fig. 3. Mean elastic modulus (E) and contact hardness (H_c_) of the cortical bone from patients with AFF were significantly higher than in the cortical bone from non‐AFF patients (Fig. 2*A*,*B*). Also, the AUC for AFF patients was greater than for non‐AFF patients (Fig. 3), corresponding to a greater plastic work of indentation (Fig. 2*E*) implying that there is an increased risk of an AFF with an increasing elastic modulus of the cortical bone. Resistance to plastic deformation of cortical bone was not significantly different between the two groups (Fig. 2*C*). After adjusting for the treatment duration, the differences between the groups remained significant. The OR for an AFF for elastic modulus of cortical bone was 1.13 (95% CI, 1.02–1.24; *p* = 0.017), and for contact hardness was 7.88 (95% CI, 1.06–58.5; *p* = 0.044; Supplemental Table S1). This suggests that an AFF is 1.13 times more likely to occur with a unit increase in elastic modulus and almost eight times more likely to occur with a unit increase in contact hardness.

There were no significant differences in any of the nanomechanical properties of cancellous bone between patients with and without an AFF (Fig. 2*A*–*E*), and none of the nanomechanical properties of cancellous bone were associated with AFF (Supplemental Table S2). However, it is interesting to note that the plastic work of indentation had a decreasing trend in cancellous bone with an AFF compared to the cortical bone (Fig. 2*E*). Indentation energy was the only nanomechanical property to exhibit this trend.

## Discussion

In this well‐characterized age‐, sex‐, race‐matched prospective nested case‐control study of postmenopausal women receiving bisphosphonate therapy, we showed that bone turnover was severely suppressed similarly in patients with and without AFF; however, the mean duration of bisphosphonate therapy was 4 years longer in patients with AFF (Table 1). This implies that although both the *degree* and *duration* of SSBT are involved in the pathogenesis of AFF, the *duration* of SSBT appears to be more important than the *degree* of suppression. In addition, we report for the first time, that wall thickness, an index of the output capacity of team of osteoblasts, was significantly lower in bone from patients with AFF than in non‐AFF patients. Thus, a combination of SSBT and impaired osteoblast team function (Table 2) may compromise bone tissue material properties as noted in Fig. 2.

Similar to our previous uncontrolled studies,^(^ ^29^, ^30^ ^)^ we have confirmed that the tissue level nanomechanical properties of bone are significantly compromised in patients on long‐term bisphosphonate therapy who sustained an AFF compared to those who did not sustain an AFF. Also, in this study we found that the nanomechanical properties were more profoundly affected in cortical bone than in cancellous bone, which may help explain why atypical fractures occur in cortical rich long tubular bones (extremities) compared to cancellous rich flat bones (vertebrae) as can be seen in some patients after discontinuation of denosumab.^(^ ^39^ ^)^

Considering the nanomechanical properties of cortical bone tissue, we found that an AFF was about 12.5% more likely to have a higher elastic modulus and AFF is eight times more likely to have occurred in bone with increased contact hardness of cortical bone. Increases in Young's modulus measured by nanoindentation may be associated with increased tissue degree of mineralization, which is consistent with our previously reported results^(^ ^29^, ^30^ ^)^ and a recent report,^(^ ^34^ ^)^ as well as in agreement with those in the literature.^(^ ^40^, ^41^ ^)^ AFF, in some respects, is a type of insufficiency stress fracture associated with accumulation of microdamage due to prolonged SSBT.^(^ ^1^, ^2^, ^9^ ^)^ Indeed, we found numerous microcracks in the cortical bone, which are known to increase with increasing mechanical loading over time and as a function of decreasing bone remodeling.^(^ ^12^, ^34^ ^)^ In addition, bisphosphonates are known to promote non‐enzymatic glycation, which increases bone fragility and disrupts microcrack toughening mechanisms in bone.^(^ ^40^, ^42^ ^)^ This study also examined the work of indentation and its potential for understanding AFF. Our results showed a trend of increased plastic indentation work in cortical bone with an AFF, although results were not significantly different from non‐AFF bone (Fig. 2*E*). It is interesting to note that the trends of plastic indentation work were different for cortical and cancellous bone; a comparative study of cortical and cancellous bone with and without an AFF might elucidate key differences associated with the underlying changes of bone subjected to long‐term bisphosphonate therapy and explain why AFFs occur.

Collectively, prolonged SSBT, reduced wall thickness, and nanoindentation results suggest that the nanomechanical properties of bone are significantly compromised in patients who have sustained an AFF. Although both the cortical and cancellous bone tissue properties are affected, the cortical bone tissue was more profoundly affected, as reflected in higher elastic modulus and contact hardness in patients with an AFF (Fig. 2*A*–*E*). Taken together, it seems reasonable to infer that atypical fractures are more common in weight‐bearing cortical‐rich long bones (femur, tibia, and metatarsals)^(^ ^1^, ^2^, ^3^, ^43^, ^44^, ^45^, ^46^ ^)^ than in non–weight‐bearing cortical‐rich bones (humerus and pubic rami).^(^ ^1^ ^)^

Of the various risk factors proposed for the development of AFFs, only the bone remodeling and mechanical properties appear to be relevant. Race/ethnicity, sex, age, and femur geometry are closely interrelated, and although femur geometry most likely determines the location of AFF (subtrochanteric versus diaphyseal), it does not necessarily cause AFF. A similar relationship might exist between height/weight and AFF, but this effect is in addition to SSBT.^(^ ^47^ ^)^ Finally, drugs known to lower bone turnover add to the risk of AFF caused by SSBT, but do not by themselves cause AFF, because no case of AFF has been reported in patients on long‐term estrogen, raloxifene, glucocorticoid, or proton‐pump inhibitor therapy alone or together without bisphosphonate therapy.

What can be inferred from our novel observations? All biologic tissues must renovate by remodeling to avoid age‐related or drug‐induced decay in tissue material properties, a process that is not unique to bone; however, the process of remodeling is critical to maintain structural integrity of bones. Thus, suppression of bone turnover over a short‐term (<5 years, for instance) may not be detrimental, but chronic suppression over prolonged periods (>5–10 years) may compromise both bone material and compositional properties.^(^ ^29^, ^30^, ^31^ ^)^ Long‐term suppression of bone turnover, especially SSBT, promotes advanced glycation of collagen, increases degree of mineralization and homogeneity of bone tissue, and decreases targeted repair of microdamage, which collectively result in bone tissue brittleness.^(^ ^48^ ^)^ Thus, chronic suppression of bone turnover appears to be the most proximate cause for the development of AFF, but the duration of suppression is more important than the degree of suppression as demonstrated in this study. This phenomenon is analogous to the development of secondary adrenal insufficiency in patients on chronic glucocorticoid therapy. A short‐term treatment, regardless of glucocorticoid dose, rarely suppresses endogenous cortisol secretion, but prolonged glucocorticoid therapy, even in small doses, results in adrenal insufficiency.^(^ ^49^, ^50^ ^)^ Accordingly, the concept of a “drug holiday” for potent anti‐resorptive therapies appears to be both rational and justified,^(^ ^51^, ^52^, ^53^ ^)^ as it is practiced for glucocorticoid therapy.

Two findings in our study deserve further exploration: the unexpected finding of reduced wall thickness and increased osteoclast surface without an increase in eroded surface. It is likely that chronic suppression of bone turnover with long‐term treatment with bisphosphonates not only *reduces osteoblast function*, but also affects *osteoclast function*, both of which are required to mitigate bone tissue microdamage accumulation, avoid increased degree of mineralization of bone,^(^ ^34^ ^)^ and prevent adverse nanomechanical properties.

Despite being a well‐designed matched nested case‐controlled study, there were a few limitations to the approach. First, the sample size was small, having been limited by the number of patients with AFF. This was not unexpected considering the rarity of AFFs and the need for an invasive transiliac bone biopsy to perform detailed bone histomorphometry and to measure nanomechanical properties. Nevertheless, we accomplished both of our objectives with novel informative findings. Second, because of the large variance in histomorphometric measurements, we may have failed to detect meaningful differences in several relevant variables such as osteoid and mineralization indices, and bone formation rate. However, the directional changes and numerical differences, although not statistically significant, suggest that SSBT may be the most proximate cause in the pathogenesis of AFF, which in turn may lead to altered bone tissue compositional properties that we did not measure. Third, we cannot exclude unintended ascertainment bias in the recruitment of patients for the study, although we did not explicitly seek specific patients. Finally, there may have been unintended confounding variables and we did not have baseline bone biopsy before initiation of bisphosphonate therapy, which may be important because further suppression of an already preexisting low bone remodeling may aggravate the situation.

Notwithstanding the limitations, our study also had several strengths. The unique study design provided useful and novel information about underlying pathogenic mechanisms for the development of AFF in women receiving long‐term bisphosphonate therapy. The current study affirms our previous uncontrolled study results and extend them by demonstrating key differences in bone histomorphometric and nanomechanical properties between patients with and without AFF. Directional changes, albeit some nonsignificant, suggest biologically plausible causality, and will need to be further investigated. The combination of bone histomorphometric and nanoindentation results suggest that cortical bone is more compromised than cancellous bone and may explain why these unusual fractures occur almost exclusively in weight‐bearing long bones such as femur, tibia, and metatarsals.

## Conclusions

Based on the current and previous studies, we conclude that long‐term BP therapy in postmenopausal women is associated with prolonged SSBT resulting in altered cortical bone tissue nanomechanical properties leading to cortical‐rich weight‐bearing long‐bone atypical fractures. The scope and magnitude of this life‐changing complication requires further clarification. And although the absolute risk is small, the relative risk is likely high considering the large number of individuals at risk because of bone density determined treatment strategy. Also, the risk of AFF during long‐term bisphosphonate therapy is probably different in younger compared to older patients and balancing the benefit/risk ratio depends heavily on treatment duration (by inference, the younger the patient, the longer the treatment duration), and remaining life expectancy (by inference the older the individual, the shorter the remaining life expectancy and greater the risk of hip fracture risk), which determines the time an individual will be at risk of sustaining an AFF.

## Conflict of Interest

All authors state that they have no conflicts of interest.

### Peer Review

The peer review history for this article is available at https://publons.com/publon/10.1002/jbm4.10523.

## Supporting information

**Supplemental Table S1** Odds ratios and 95% confidence intervals (95% CI) with bisphosphonate treatment duration and nanomechanical properties of cortical bone as predictive variables for atypical femur fracture.
**Supplemental Table S2** Odds ratios and 95% confidence intervals (95% CI) with bisphosphonate treatment duration and nanomechanical properties of cancellous bone as predictive variables for atypical femur fracture.
