# Supplementary material for: Bone Nanomechanical Properties and Relationship to Bone Turnover and Architecture in Patients With Atypical Femur Fractures: A Prospective Nested Case‐Control Study
Source: JBMR Plus. 2021 Aug 12;5(9):e10523. doi: 10.1002/jbm4.10523 (PMC8441274; doi:10.1002/jbm4.10523)
Supplement: Supplementary file 1 — Supplemental Table S1 Odds ratios and 95% confidence intervals (95% CI) with bisphosphonate treatment duration and nanomechanical properties of cortical bone as predictive variables for atypical femur fracture. Supplemental Table S2 Odds ratios and 95% confidence intervals (95% CI) with bisphosphonate treatment duration and nanomechanical properties of cancellous bone as predictive variables for atypical femur fracture. [file JBM4-5-e10523-s001.docx]

**Supplemental Table-1:** Odds Ratios and 95% confidence intervals (95% CI) with bisphosphonate treatment duration and nanomechanical properties of cortical bone as predictive variables for Atypical Femur Fracture.

| **Parameter** | **Odds Ratio** | **95 % CI** | | **p** |
| --- | --- | --- | --- | --- |
| Treatment Duration, y | 1.189 | 0.992 | 1.425 | 0.062 |
| E, GPa | 1.125 | 1.021 | 1.240 | 0.017 |
|  |  |  |  |  |
| Treatment Duration, y | 1.178 | 0.976 | 1.422 | 0.087 |
| Resistance to Plastic Deformation, H, GPa | 1.128 | 0.776 | 1.640 | 0.527 |
|  |  |  |  |  |
| Treatment Duration, y | 1.183 | 0.981 | 1.426 | 0.078 |
| Contact Hardness, Hc, GPa | 7.883 | 1.062 | 58.498 | 0.044 |
|  |  |  |  |  |
| Treatment Duration, y | 1.177 | 0.976 | 1.420 | 0.088 |
| Elastic Indentation Work, Ue, nJ | 1.528 | 0.021 | 112.618 | 0.847 |
|  |  |  |  |  |
| Treatment Duration, y | 1.179 | 0.978 | 1.422 | 0.084 |
| Plastic Indentation Work, Up, nJ | 2.397 | 0.472 | 12.172 | 0.292 |

**Supplemental Table-2:**  Odds Ratios and 95% confidence intervals (95% CI) with bisphosphonate treatment duration and nanomechanical properties of cancellous bone as predictive variables for Atypical Femur Fracture.

| **Parameter** | **Odds Ratio** | **95 % CI** | | **p** |
| --- | --- | --- | --- | --- |
| Treatment Duration, y | 1.202 | 0.986 | 1.465 | 0.069 |
| E, GPa | 1.010 | 0.913 | 1.117 | 0.846 |
|  |  |  |  |  |
| Treatment Duration, y | 1.200 | 0.985 | 1.462 | 0.070 |
| Resistance to Plastic Deformation, H, GPa | 1.381 | 0.958 | 1.993 | 0.084 |
|  |  |  |  |  |
| Treatment Duration, y | 1.200 | 0.986 | 1.462 | 0.069 |
| Contact Hardness, Hc, GPa | 3.564 | 0.377 | 33.710 | 0.268 |
|  |  |  |  |  |
| Treatment Duration, y | 1.203 | 0.986 | 1.467 | 0.069 |
| Elastic Indentation Work, Ue, nJ | 2.928 | 0.116 | 74.091 | 0.515 |
|  |  |  |  |  |
| Treatment Duration, y | 1.199 | 0.983 | 1.464 | 0.074 |
| Plastic Indentation Work, Up, nJ | 0.601 | 0.120 | 3.018 | 0.537 |
